# 

**Published:** 1889-04

**Authors:** 


### THE MEDICAL ASSOCIATION OF GEORGIA.

The Association will hold its fortieth annual session in Macon, April 17-19, prox. The present outlook gives promise of an exceedingly interesting and profitable meeting. We are confidently looking to the Committee on Programme to work up a busy session. It is earnestly hoped that every member of the Association will feel individually concerned, and will lend his aid in infusing new life into the Association.

The local committee of arrangements are perfecting plans for three busy *days*, while the *evenings* alone will be devoted to recreation and pleasure. Macon will give the Association a cordial greeting, and hopes to have a large attendance.

All the railroads of the State will give reduced rates. Be sure to secure from the local agent at the starting point certificate of purchase of going ticket, which certificate, after being signed by the Secretary, will entitle you to purchase return ticket at one-third the regular rates. This applies to all the roads in the State, except the Georgia road and its branches. On these, the Secretary will be furnished with certificate of attendance, which will entitle you to return on one-third fare.

A cordial and pressing invitation is extended to every doctor in Georgia, "of our faith and order," to attend the approaching session and join the Association. Let every member "put his shoulder to the wheel," and make the next session the most interesting in the history of the Association.

K. P. MOORE, Secretary.

